# The potential of combined mutation sequencing of plasma circulating cell‐free DNA and matched white blood cells for treatment response prediction

**DOI:** 10.1002/1878-0261.12646

**Published:** 2020-02-23

**Authors:** Paul van der Leest, Ed Schuuring

**Affiliations:** ^1^ Laboratory of Molecular Pathology Department of Pathology (EA10) University Medical Center Groningen University of Groningen Groningen The Netherlands

## Abstract

Highly sensitive mutation detection methods enable the application of circulating cell‐free DNA for molecular tumor profiling. Recent studies revealed that sequencing artifacts, germline variants, and clonal hematopoiesis confound the interpretation of sequencing results and complicate subsequent treatment decision making and disease monitoring. Parallel sequencing of matched white blood cells promises to overcome these issues and enables appropriate variant calling.

Comment on: https://doi.org/10.1002/1878-0261.12617

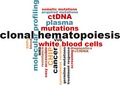

**Abbreviations**
CHIP: clonal hematopoiesis of indeterminate potential
ctDNA: circulating tumor DNA
ccfDNA: circulating cell-free DNA
ER: estrogen receptor
MBC: metastatic breast cancer
PFS: progression‐free survival
WBC: white blood cells

Circulating cell‐free DNA (ccfDNA) in the plasma of cancer patients constitutes a potential source of tumor‐derived DNA. Sensitive mutation detection assays on ccfDNA extracted from plasma could be used to detect circulating tumor DNA (ctDNA). This poses opportunities to apply ctDNA as an easily accessible biomarker for cancer screening, predictive testing, and monitoring of disease and treatment responses (Heitzer *et al.*, 2019; Lampignano *et al.*, 2019; Pantel and Alix‐Panabières, 2019). However, blood‐based molecular tumor profiling has been approached with caution since the origin of the detected variants is uncertain. CcfDNA consists of mostly degraded DNA fragments shedded from various tissues through apoptosis, necrosis, exocytose, or active secretion, of which over 90% derives from the hematological lineage (Abbosh, Birkbak, and Swanton, 2018; Thierry *et al.*, 2016; Xia *et al.*, 2017). High‐sensitive mutation detection methods using ccfDNA from cancer patients demonstrated a low overall yield of total DNA and that the ctDNA fraction accounts only for a very small proportion of the total ccfDNA of less than 0.1–1%. This fraction varies significantly according stage of disease, response to treatment, tumor burden, and tumor characteristics such as tumor grade, vascularization, cell death, and proliferation rates (Heitzer *et al.*, 2019). Since the ctDNA fraction is extremely low in many cancers, ctDNA detection methods are required to be highly sensitive and highly specific (Elazezy and Joosse, 2018; Merker *et al.*, 2018). Recent developments in high‐sensitive, more sophisticated sequencing methodologies to detect tumor‐derived mutations in ctDNA enabled to identify variants that are present at very low levels in a background of ‘normal’ ccfDNA using, for example, combinations of integrated digital error suppression (like unique‐molecular‐identifier), appropriate variant calling, multigene analysis, and in‐depth sequencing (Abbosh *et al.*, 2018; Heitzer *et al.*, 2019; Razavi *et al.*, 2019).

In this issue, Kruger and colleagues determined the presence of hotspot mutations and ctDNA load using a high‐sensitive sequencing 10‐gene panel approach to describe treatment outcome in estrogen receptor (ER)‐positive, HER2‐negative metastatic breast cancer (MBC) patients treated with everolimus and exemestane (EVE/EXE) (Kruger *et al.*, 2020). In this study, 76% of the included MBC patients were considered ctDNA positive with a high prevalence of ESR1, PIK3CA, and TP53 variants. A shorter progression‐free survival (PFS) was found in patients with three or more mutations (*P* = 0.003) or with 54 or more mutant ctDNA copies (*P* = 0.002). A recent study on a comparable cohort showed similar associations between high quantities of ctDNA and a diminished survival (Suppan *et al.*, 2019). The study of Kruger and colleagues is one of the first to demonstrate the potential of ctDNA mutation testing using pretreatment plasma to select patients with ER‐positive/HER2‐negative MBC eligible for EVE/EXE with prolonged PFS and that high‐sensitive sequencing of ccfDNA might support predicting treatment response in MBC.

In addition, the analysis revealed that certain (likely) pathogenic mutations in ESR1 and SF3B1 might affect PFS and OS as well (*P* = 0.084 and *P* = 0.088). In line with previous reports, specific ESR1 mutations such as Y537S were considered as adverse prognostic biomarkers while other mutations, like in PIK3CA, do not affect PFS (Moynahan *et al.*, 2017; Reinert *et al.*, 2017). Besides, in a similar cohort using a larger gene panel, other specific mutations in AR, MUC16, and ERBB2 (not tested in the Kruger study) revealed that each separately had a significant association with survival in MBC (Keup *et al.*, 2019). These findings imply that not just the number of observed different hotspot mutations might be associated with treatment response in MBC, but the presence or absence of certain strongly pathogenic mutations such as ESR1 or MUC16 might influence survival significantly. Therefore, future experiments of larger cohorts are needed to evaluate the contribution of these separate pathogenic mutations in combination with the total number of other mutations on clinical outcome to further improve the value of ctDNA testing as a predictive biomarker for survival.

An important drawback of the implementation of innovative high‐sensitive ccfDNA sequencing approaches is the detection of variants that are not derived from the vital tumor cells. Some of these variants are the result of technical artifacts during ccfDNA sequence analysis. This was recently illustrated when comparing 4 different commercially available next‐generation sequencing methodologies with considerable high discordances reflected in many false‐positive and false‐negative results (Stetson *et al.*, 2019). Other insignificant variants appear due to inappropriate variant calling resulting from inaccurate discrimination of somatic tumor‐relevant variants from SNPs, germ‐line mutations, sequencing artifacts, clonal hematopoiesis of indeterminate potential (CHIP) among others. All these inappropriate variant callings may confound the interpretation of ccfDNA sequencing in particular when applied to investigate associations with tumor response and clinical outcome.

Clonal hematopoiesis of indeterminate potential is the consequence of the accumulation of somatic mutations resulting from replication errors in the rapidly dividing and mutation‐prone hematopoietic progenitors (Gondek and DeZern, 2020; Razavi *et al.*, 2019). These somatic mutations may provide a selective benefit to some hematopoietic stem cells and their progenitors, resulting in their disproportionate expansion. Since the majority of ccfDNA is blood cell‐derived, somatic mutations associated with CHIP can thus be detected during ccfDNA sequencing analysis (Gondek and DeZern, 2020; Razavi *et al.*, 2019). Indeed, Chen and coworkers detected somatic mutations in the ccfDNA in 30% of healthy aging individuals in genes related to hematological malignancies including TP53 (Chen *et al.*, 2019). Razavi and associates using high‐intensity sequencing with 401‐gene panel reported that most somatic mutations detected in control patients without cancer (81.6%) were also identified in their matched white blood cells (WBC) (Razavi *et al.*, 2019). Similarly, most mutations identified in ccfDNA samples of cancer patients (including MBC) were also found in their matched WBC (53.2%). Furthermore, the number of WBC‐matched ccfDNA variants in cancer patients did not correlate with the number of tumor biopsy‐matched mutations. All these specific somatic mutations are less likely to be of tumor origin and have features consistent with CHIP (Razavi *et al.*, 2019).

In summary, the recent achievements in high‐sensitive sequencing methodologies of pretreatment plasma ccfDNA have proven to become an useful tool to detect and map tumor‐derived mutations and offer opportunities as those reported by Kruger and colleagues, to investigate the clinical value for the prediction of therapy response and clinical outcome. However, these same high‐sensitive sequencing methodologies now also visualize that most variants detected in ccfDNA of cancer patients represent especially CHIP and that CHIP is more prevalent than was previously anticipated (Chen *et al.*, 2019; Razavi *et al.*, 2019). In particular, this high prevalence of CHIP emphasizes the importance of parallel high‐sensitive sequencing of DNA derived from WBCs of the same patient for appropriate variant interpretation.

## Conflict of interest

PvdL has no conflicts of interest to declare. ES received honoraria for advisory board from AstraZeneca, Roche, Pfizer, Bayer, Novartis, BMS, BioRad, Illumina, Ageno BioSciences, Janssen Cilag (Johnson&Johnson), BioCartis; speaker’s fee from AstraZeneca, Roche, Pfizer, Novartis, BioRad, Illumina, BioCartis; and research support from Boehringer Ingelheim, BMS, Biocartis, Bio‐Rad, Ageno BioSciences, and Roche (all outside the submitted work and all fees to UMCG).

## References

[mol212646-bib-0001] Abbosh C , Birkbak NJ and Swanton C (2018) Early stage NSCLC — challenges to implementing ctDNA‐based screening and MRD detection. Nat Rev Clin Oncol 15, 577–586. 29968853 10.1038/s41571-018-0058-3

[mol212646-bib-0002] Chen S , Wang Q , Yu H , Capitano ML , Vemula S , Nabinger SC , Gao R , Yao C , Kobayashi M , Geng S *et al* (2019) Mutant p53 drives clonal hematopoiesis through modulating epigenetic pathway. Nat Commun 10, 5649. 31827082 10.1038/s41467-019-13542-2 PMC6906427

[mol212646-bib-0003] Elazezy M and Joosse SA (2018) Techniques of using circulating tumor DNA as a liquid biopsy component in cancer management. Comput Struct Biotechnol J 16, 370–378. 30364656 10.1016/j.csbj.2018.10.002 PMC6197739

[mol212646-bib-0004] Gondek LP and DeZern AE (2020) Assessing clonal haematopoiesis: clinical burdens and benefits of diagnosing myelodysplastic syndrome precursor states. Lancet Haematol 7, e73–e81. 31810765 10.1016/S2352-3026(19)30211-X PMC7008978

[mol212646-bib-0005] Heitzer E , Haque IS , Roberts CES and Speicher MR (2019) Current and future perspectives of liquid biopsies in genomics‐driven oncology. Nat Rev Genet 20, 71–88. 30410101 10.1038/s41576-018-0071-5

[mol212646-bib-0006] Keup C , Benyaa K , Hauch S , Sprenger‐Haussels M , Tewes M , Mach P , Bittner AK , Kimmig R , Hahn P and Kasimir‐Bauer S (2019) Targeted deep sequencing revealed variants in cell‐free DNA of hormone receptor‐positive metastatic breast cancer patients. Cell Mol Life Sci 77, 497–509. 31254045 10.1007/s00018-019-03189-z PMC7010653

[mol212646-bib-0007] Kruger DT , Jansen MPHM , Konings IRHM , Dercksen WM , Jager A , Oulad Hadj J , Sleijfer S , Martens JWM and Boven E (2020) High ctDNA molecule numbers relate with poor outcome in advanced ER+, HER2− postmenopausal breast cancer patients treated with everolimus and exemestane. Mol Oncol, 14, 490–503. 10.1002/1878-0261.12617 PMC7053245 31841262

[mol212646-bib-0008] Lampignano R , Neumann MHD , Weber S , Voss T , Groelz D , Babayan A , Schlumpberger M , Chemi F , Wikman H , Galizzi JP *et al* (2019) Multicenter evaluation of ccfDNA extraction and downstream analyses for the development of standardized (pre)analytical workflows. Clin Chem 66, 1. 10.1373/clinchem.2019.306837 31628139

[mol212646-bib-0009] Merker JD , Oxnard GR , Compton C , Diehn M , Hurley P , Lazar AJ , Lindeman N , Lockwood CM , Rai AJ , Schilsky RL *et al* (2018) Circulating tumor DNA analysis in patients with cancer: american society of clinical oncology and college of american pathologists joint review. J Clin Oncol 36, 1631–1641. 29504847 10.1200/JCO.2017.76.8671

[mol212646-bib-0010] Moynahan ME , Chen D , He W , Sung P , Samoila A , You D , Bhatt T , Patel P , Ringeisen F , Hortobagyi GN *et al* (2017) Correlation between PIK3CA mutations in cell‐free DNA and everolimus efficacy in HR+, HER2‐ advanced breast cancer: results from BOLERO‐2. Br J Cancer 116, 726–730. 28183140 10.1038/bjc.2017.25 PMC5355930

[mol212646-bib-0011] Pantel K and Alix‐Panabières C (2019) Liquid biopsy and minimal residual disease — latest advances and implications for cure. Nat Rev Clin Oncol 16, 409–424. 30796368 10.1038/s41571-019-0187-3

[mol212646-bib-0012] Razavi P , Li BT , Brown DN , Jung B , Hubbell E , Shen R , Abida W , Juluru K , De Bruijn I , Hou C *et al* (2019) High‐intensity sequencing reveals the sources of plasma circulating cell‐free DNA variants. Nat Med 25, 1928–1937. 31768066 10.1038/s41591-019-0652-7 PMC7061455

[mol212646-bib-0013] Reinert T , Saad ED , Barrios CH and Bines J (2017) Clinical implications of ESR1 mutations in hormone receptor‐positive advanced breast cancer. Front Oncol 7, 26. 28361033 10.3389/fonc.2017.00026 PMC5350138

[mol212646-bib-0014] Stetson D , Ahmed A , Xu X , Nuttall BRB , Lubinski TJ , Johnson JH , Barrett JC and Dougherty BA (2019) Orthogonal comparison of four plasma NGS tests with tumor suggests technical factors are a major source of assay discordance. JCO Precis Oncol 3, 1–9. 10.1200/PO.18.00191 35100678

[mol212646-bib-0015] Suppan C , Brcic I , Tiran V , Mueller HD , Posch F , Auer M , Ercan E , Ulz P , Cote RJ , Datar RH *et al* (2019) Untargeted assessment of tumor fractions in plasma for monitoring and prognostication from metastatic breast cancer patients undergoing systemic treatment. Cancers 11, 1171. 10.3390/cancers11081171 PMC6721524 31416207

[mol212646-bib-0016] Thierry AR , El Messaoudi S , Gahan PB , Anker P and Stroun M (2016) Origins, structures, and functions of circulating DNA in oncology. Cancer Metastasis Rev 35, 347–376. 27392603 10.1007/s10555-016-9629-x PMC5035665

[mol212646-bib-0017] Xia L , Li Z , Zhou B , Tian G , Zeng L , Dai H , Li X , Liu C , Lu S , Xu F *et al* (2017) Statistical analysis of mutant allele frequency level of circulating cell‐free DNA and blood cells in healthy individuals. Sci Rep 7, 7526. 28790338 10.1038/s41598-017-06106-1 PMC5548860

